# Eleven Cases of Resection of Knee Operated with Success after Suppurating Arthritis

**Published:** 1918-12

**Authors:** 


***Eleven Cases of Resection of the Knee Operated with Success after Suppurating Arthritis.*** By Dr. TRIDON. *Bulletin de la Société de Chirurgie de Paris.* April, 30, 1918.

The author has observed eleven cases of resection of the knee. They all have given good results. He insists on the advantages of operating as soon as possible.

He removes the patella, cleanses the soft parts and the injured bones, and completely excises the synovial membrane. He does not consider it necessary to do a suture of the fragments, but wraps the whole limb up in plaster and fastens it with appropriate splints. After three months, the men begin walking about without an apparatus. The shortening of the leg does not exceed 3 or 4 cm.

These observations were made in 1915 and 1916. Since then great progress has been made in the treatment of injuries of the joints, especially by the complete cleansing of the wound, with excision of all bruised tissues; and very favorable results can be obtained in that way without operation. But Dr. Tridon has shown that the resection of the knee is quite possible when other treatments have failed to prevent suppurating arthritis.

